# Persistent polypharmacy and fall injury risk: the Health, Aging and Body Composition Study

**DOI:** 10.1186/s12877-021-02695-9

**Published:** 2021-12-15

**Authors:** Lingshu Xue, Robert M. Boudreau, Julie M. Donohue, Janice C. Zgibor, Zachary A. Marcum, Tina Costacou, Anne B. Newman, Teresa M. Waters, Elsa S. Strotmeyer

**Affiliations:** 1 Department of Epidemiology, Graduate School of Public Health, University of Pittsburgh, 130 N Bellefield Avenue, Suite 300, Pittsburgh, PA 15213 USA; 2 Department of Health Policy and Management, Graduate School of Public Health, University of Pittsburgh, Pittsburgh, PA USA; 3 College of Public Health, University of South Florida, Tampa, FL 33612 USA; 4 Department of Pharmacy, School of Pharmacy, University of Washington, Seattle, WA USA; 5 Department of Health Management and Policy, University of Kentucky College of Public Health, Lexington, KY USA

**Keywords:** Persistent polypharmacy, Treated fall injury, Medicare, Medication, Falls, FRID, Geriatrics

## Abstract

**Background:**

Older adults receive treatment for fall injuries in both inpatient and outpatient settings. The effect of persistent polypharmacy (i.e. using multiple medications over a long period) on fall injuries is understudied, particularly for outpatient injuries. We examined the association between persistent polypharmacy and treated fall injury risk from inpatient and outpatient settings in community-dwelling older adults.

**Methods:**

The Health, Aging and Body Composition Study included 1764 community-dwelling adults (age 73.6 ± 2.9 years; 52% women; 38% black) with Medicare Fee-For-Service (FFS) claims at or within 6 months after 1998/99 clinic visit. Incident fall injuries (*N* = 545 in 4.6 ± 2.9 years) were defined as the initial claim with an ICD-9 fall E-code and non-fracture injury, or fracture code with/without a fall code from 1998/99 clinic visit to 12/31/08. Those without fall injury (*N* = 1219) were followed for 8.1 ± 2.6 years. Stepwise Cox models of fall injury risk with a time-varying variable for persistent polypharmacy (defined as ≥6 prescription medications at the two most recent consecutive clinic visits) were adjusted for demographics, lifestyle characteristics, chronic conditions, and functional ability. Sensitivity analyses explored if persistent polypharmacy both with and without fall risk increasing drugs (FRID) use were similarly associated with fall injury risk.

**Results:**

Among 1764 participants, 636 (36%) had persistent polypharmacy over the follow-up period, and 1128 (64%) did not. Fall injury incidence was 38 per 1000 person-years. Persistent polypharmacy increased fall injury risk (hazard ratio [HR]: 1.31 [1.06, 1.63]) after adjusting for covariates. Persistent polypharmacy with FRID use was associated with a 48% increase in fall injury risk (95%CI: 1.10, 2.00) vs. those who had non-persistent polypharmacy without FRID use. Risks for persistent polypharmacy without FRID use (HR: 1.22 [0.93, 1.60]) and non-persistent polypharmacy with FRID use (HR: 1.08 [0.77, 1.51]) did not significantly increase compared to non-persistent polypharmacy without FRID use.

**Conclusions:**

Persistent polypharmacy, particularly combined with FRID use, was associated with increased risk for treated fall injuries from inpatient and outpatient settings. Clinicians may need to consider medication management for FRID and other fall prevention strategies in community-dwelling older adults with persistent polypharmacy to reduce fall injury risk.

**Supplementary Information:**

The online version contains supplementary material available at 10.1186/s12877-021-02695-9.

## Background

Medication use may be associated with falls and fall injuries among older adults. Nearly one third of community-dwelling U.S. adults over 65 years old experience falls annually, with the percent increasing to 50% in adults over 80 years old [1, 2]. Of those who fall, 5% incur serious injuries such as fractures, joint dislocation, and head trauma [3]. Each year, nearly 3 million older adults receive treatment for fall injuries in emergency departments (ED), and around 800,000 are hospitalized for fall injuries [4]. In the US, fall injuries resulted in approximately $668 billion in medical expenses during 2015 [5]. One potentially modifiable risk factor for fall injury is multiple medication use, commonly referred to as polypharmacy [6]. In previous studies related to fall injury, polypharmacy has been defined according to the number of medications measured in a single short period (e.g. 90 days) [7–9]. However, older adults may vary in use of medications over time [10, 11]. Newly diagnosed acute or chronic conditions may prompt an increase in the number of medications, while deprescribing and poor adherence may reduce the number [11]. Considering whether polypharmacy was persistent or transient over time may be important to identify older adults at high risk of fall injury.

The role of persistent polypharmacy in fall injury remains largely unexamined. In a few previous studies based on claims data, persistent polypharmacy was defined as use of multiple medications for more than 6 months in a year follow-up; however many older adults have polypharmacy for longer duration and additionally these studies did not consider fall injury risks [12, 13]. Studies examining polypharmacy persisting longer than 1 year are strongly needed. One study of adults aged 60-64 years found that polypharmacy at two time points 5 years apart doubled the decline in chair rise speed compared to polypharmacy only at one time point [14], suggesting persistent polypharmacy for more than 1 year may be associated with reduced physical function. However, this study did not account for comorbid conditions or factors related to physical function in the analysis, and did not investigate fall injury outcomes [14]. To fill this gap, our study defined persistent polypharmacy as use of multiple medications in two consecutive years based on our previous studies in this cohort [15, 16], and examined its association with fall injury. In addition, fall-risk increasing drugs (FRID, i.e. sedatives and hypnotics, neuroleptics and antipsychotics, antidepressants, benzodiazepines, and opioids) specifically appear to be strongly related to fall injuries rather than polypharmacy alone [17–19]. Whether persistent polypharmacy with FRID use is associated with a higher fall injury risk compared to polypharmacy without FRID use has not been well investigated. Understanding the role of persistent polypharmacy in fall injury is important for evaluating the appropriateness of deprescribing and providing a tailored intervention to reduce fall injury risk in older adults. Therefore, our objective was to determine whether persistent polypharmacy, both with and without FRID use, is associated with an increased risk of fall injuries treated in inpatient and outpatient settings among community-dwelling older adults with Medicare Fee-for-service (FFS).

## Methods

### Study design and participants

**Fig. 1:**
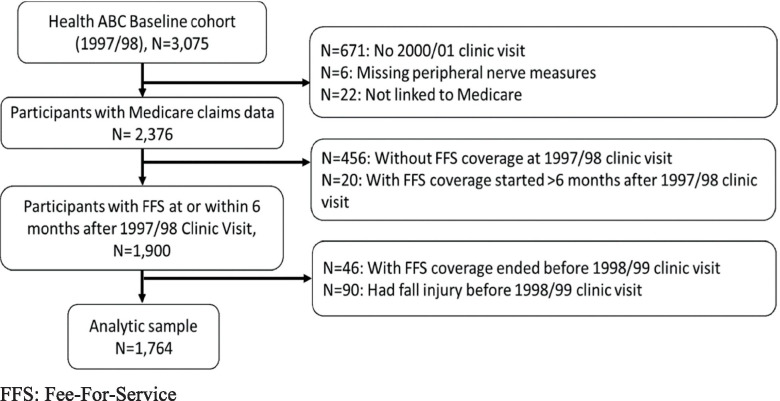
Flow chart displaying selection of study participants for analysis. FFS: Fee-For-Service

The Health, Aging and Body Composition (Health ABC) Study is a prospective cohort study designed to investigate if changes in body composition act as a common pathway by which multiple diseases affect morbidity, disability, and risk of mortality in community-dwelling older adults [20]. This study included 3075 participants (48.4% male, 41.6% black) aged 70–79 years at baseline (1997/1998) [20]. They were recruited from a random sample of Medicare beneficiaries in Memphis, Tennessee (TN), and Pittsburgh, Pennsylvania (PA), and all age-eligible black community residents in these areas [21]. Participants were excluded if they: 1) reported difficulty with walking one-quarter of a mile or climbing 10 steps without resting, or difficulty with performing mobility-related activities of daily living; 2) had life-threatening cancers and received active treatment in the past 3 years; or 3) had plans of leaving the area within 3 years [21]. The institutional review boards at the participating institutions approved the study protocol, and written informed consent was obtained before testing and data collection [21]. The exclusion criteria for this analysis included 1) missing a 2000/01 clinic visit (*N* = 671) since Medicare data was only linked for those participants; 2) lacking linked Medicare FFS Parts A and B claim data (*N* = 28); 3) not having FFS coverage at 1997/98 baseline clinic visit (*N* = 456) or within 6 months after the baseline visit (*N* = 20); and 4) having fall injury (*N* = 90) or ending FFS coverage (*N* = 46) before 1998/99 clinic visit when persistent polypharmacy was measured (Fig. 1). After exclusions, the analytic sample included 1764 participants.

### Primary independent variables

**Fig. 2:**
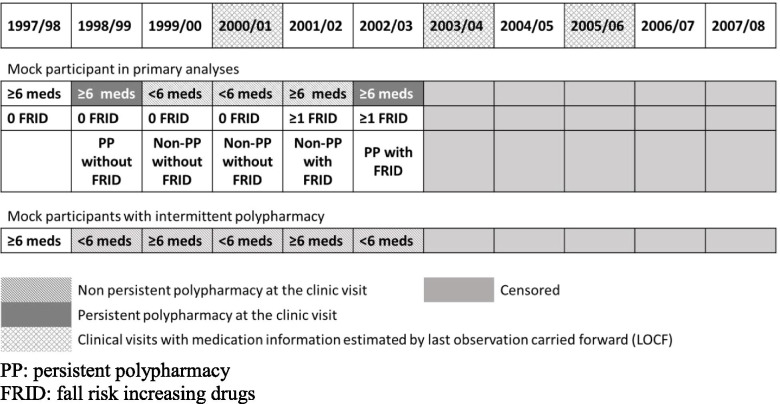
Timeline and illustrations for persistent polypharmacy. PP: persistent polypharmacy. FRID: fall risk increasing drugs

The primary independent variable was a time-varying indicator of persistent polypharmacy. We defined persistent polypharmacy as use of ≥6 prescription medications at both of the two most recent consecutive annual clinic visits based on the established Health ABC definition for the long-term use of fall-related medications [6, 15]. Compared to some definitions for polypharmacy, which define it as ≥5 medications in short periods, we used a cut-point of ≥6 medications due to known increase in the number of medications taken as age increases, especially considering that polypharmacy was measured as time-varying in our cohort with more than 8 years follow-up. Non-persistent polypharmacy was defined as use of ≥6 medications only at one visit or use of <6 medications at both of the two most recent consecutive visits. Participants with intermittent polypharmacy (i.e. using ≥6 medications at some clinic visits but never having persistent polypharmacy during the entire study period, *N* = 174) were included only in the sensitivity analyses to distinguish the effect of persistent polypharmacy from that of polypharmacy at a single time point. FRID include antiepileptic drugs, hypnotics/sedatives drugs, antipsychotics, antidepressants, benzodiazepines, and opioids based on previous reviews for FRID and the 2015 updates of the Beers’ criteria (supplementary Table S1) [17–19]. Antihypertensives and anticholinergic drugs (except for antidepressants and antipsychotics) were not included due to non-significant association with fall risk shown in previous published papers in the Health ABC Study [16, 22]. A time-varying FRID use indicator was created for sensitivity analyses based on if participants used ≥1 FRID at most recent (short-term) or both (long-term) of the two most recent consecutive visits since a previous publication in our study showed both short-term and long-term use of FRID were related to increased fall risk [15] (Fig. 2).

Medication information was collected at clinic visits in 1997/98, 1998/99, 1999/2000, 2001/02, 2002/03, 2004/05, 2006/07, and 2007/08 and coded according to the Iowa Drug Information System [23]. Participants brought all medications taken during the 2 weeks before the clinic visit. Trained researchers transcribed medication information from the containers. Non-prescription medications such as over-the-counter medications, and herbal and dietary supplements are not commonly included in the definition of polypharmacy [6] so we did not include these. Since medication information was not collected in 2000/01, 2003/04, and 2005/06, we used the method of last observation carried forward (LOCF) to estimate the missing data. Sensitivity analyses also were conducted in the data without the estimation by LOCF.

### Outcome measure

The primary outcome was incident treated fall injury from the 1998/99 clinic visit to 12/31/08, identified using Fee-for-Service (FFS) Part A and Part B Medicare claim datasets which included inpatient, outpatient, physician/supplier, and carrier (claims from non-institutional providers) files [21]. The Medicare claims allowed fall injury events treated in inpatient and outpatient settings (e.g. inpatient hospitals, outpatient clinics, and emergency departments) to be collected if participants did not attend clinic visits or provide self-report data [21]. ICD-9 E-codes (E880-888) which describe the “mechanism of injury” were used to ascertain falls, and ICD-9 billing codes were used to identify injuries and fractures [21]. Incident fall injury was defined as the first event with 1) an ICD-9 fall E-code (E880-888) and non-fracture injury, 2) a vertebral fracture code (805-806) with a fall E-code, or 3) a non-vertebral fracture code (800-804, 807-829) with/without a fall E-code [21]. We excluded all traumatic injuries (e.g., motor vehicle accidents), intentional injuries, and pathologic injuries [21]. We compared a subset of Medicare fall injuries to the self-reported fall injuries with medical records to validate our diagnoses code algorithm which showed excellent agreement with the medical records [21]. We excluded vertebral fractures without fall codes in our algorithm since only 50% of vertebral fractures in Medicare claims were fall-related when validated with medical records [21]. Participants were followed until the first occurrence of a fall injury, the end of FFS coverage, loss to follow-up, death, or through the end of 2008 when Medicare claims were last collected for the study.

### Covariates

All covariates were measured at the 1997/98 clinic visit unless noted otherwise. Self-reported demographic variables included age, sex, race (white or black), education (<high school (HS), HS graduate, or postsecondary), and study site (Memphis or Pittsburgh) [21]. Height was measured using a stadiometer and weight was measured on a calibrated balance beam scale [21]. The baseline BMI was calculated using the formula of weight (kilograms) divided by height (meters) squared [21]. Lifestyle factors included alcohol consumption (> 1 drink/week), self-reported physical activity (kcal/kg per week spent walking, climbing stairs, and exercise), and self-reported smoking status (current, past, never; at the 1999/2000 clinic visit) [24].

Participants self-reported cardiovascular disease (i.e. bypass or coronary artery bypass graft, carotid endarterectomy, myocardial infarction, angina pectoris, or congestive heart failure), cerebrovascular disease (i.e. stroke or transient ischemic attack), poor vision (poor eyesight, cataract, glaucoma, retinal disease, and/or macular degeneration), history of falls, knee pain for > 1 month, and leg pain when walking. We defined diabetes as self-reported physician diagnosis, use of hypoglycemic medication, or fasting glucose ≥126 mg/dL (47.0 mmol/L) after an 8-h fast [25]. Diagnosed and/or treated hypertension (“hypertension” hereafter) was defined through a study-based definition as self-reported hypertension with use of any antihypertensive medications, or sitting systolic blood pressure ≥ 140 mmHg or/and sitting diastolic blood pressure ≥ 90 mmHg [21, 22]. Depressive symptoms were assessed based on the Center for Epidemiologic Studies Depression Scale (CES-D) [26]. Cognitive function was measured by the Modified Mini-Mental State Examination (3MSE) score [27]. Poor renal function was defined as cystatin C level > 1 mg/dL [28]. Sensory nerve impairment was defined as insensitivity to 1.4-g monofilament at the 2000/01 clinic visit [29]. We also included physical performance as 6-m usual gait speed [30].

### Statistical analyses

We compared baseline characteristics for those with vs. without fall injury using t-tests for normally distributed variables, Wilcoxon rank-sum tests for non-normally distributed variables, and Chi-squared tests for categorical variables. Because the status of persistent polypharmacy varied over time, we reported the event rate of persistent polypharmacy in the follow-up as well as fall injury incidence rates in the periods of persistent polypharmacy and non-persistent polypharmacy. We also included the indicators of persistent polypharmacy as a time-varying predictor in multivariable Cox proportional hazards (Cox PH) regression to estimate fall injury risk from the 1998/99 clinic visit. Baseline covariates described above were time-independent and added to the model in a stepwise manner. To minimize collinearity, additional covariates except for age, sex, and race were subsequently removed at a *P* > 0.10 and if the effect of persistent polypharmacy was not modified > 10% [31]. In sensitivity analyses, we included a combination time-varying variable for persistent polypharmacy with and without FRID use in models, defined as: persistent polypharmacy with FRID use; persistent polypharmacy without FRID use; non-persistent polypharmacy with FRID use; non-persistent polypharmacy without FRID use [19]. Analyses were done in the cohort including 174 participants with intermittent polypharmacy (*N* = 1764). In addition to the cut-point of 6 medications in the primary analyses, we used 2 to 5 and 7 to 10 as alternative cut-points to define persistent polypharmacy (e.g. ≥2 vs. < 2 medication, ≥3 vs. < 3 medications, ≥4 vs. < 4 medications, ≥5 vs. < 5 medications, etc.) and repeated the analyses [7–9]. All analyses were repeated without LOCF to check for consistency of results. No violation of PH assumption was detected. Analyses were performed using SAS, version 9.4 (SAS Institute, Inc., Cary, NC).

## Results

At the 1997/98 clinic visit, participants were aged 73.6 ± 2.9 years, 53% were women, and 37% were black. In the analytic sample (*N* = 1764), participants were followed for 8.0 ± 3.1 years from the 1998/99 clinic visit. Among 1764 participants, 36% (*N* = 636) had persistent polypharmacy, and 64% (*N* = 1128) did not. The rate of persistent polypharmacy was 179 events per 1000 person-years. Incident fall injuries occurred in 31% of 1764 participants (*N* = 545, 414 fracture and 131 non-fracture) over 4.6 ± 2.9 years following the 1998/99 clinic visit. Those without fall injury (*N* = 1219) were followed for 8.1 ± 2.6 years. The incident rate of fall injury was 38 per 1000 person-years in the follow-up, with 50 per 1000 person-year during the period of persistent polypharmacy, and 36 per 1000 person-year during the period of non-persistent polypharmacy. Participants with fall injury were older and more likely to be women, white, and a high school graduate compared to those without fall injury (Table 1). Participants with fall injuries were more likely to have lower BMI, higher 3MSE scores, and self-report a fall in the past 12 months. No difference was found between those with and without fall injuries for diabetes, cardiovascular disease, cerebrovascular disease, CES-D scores, poor vision, knee or leg pain, gait speed, 1.4-g monofilament insensitivity, or cystatin C level > 1 mg/dL. No differences were seen in the results of descriptive analyses between the samples including (*N* = 1764) and excluding those with intermittent polypharmacy (*N* = 1590).

**Table 1:** Baseline characteristics and medication use over time by incident fall injury status (*N* = 1764)

Characteristics, Mean ± SD or N (%)	Fall injury (*N* = 545)	No Fall injury (*N* = 1219)
Age, years	73.9 ± 3.0^a^	73.5 ± 2.8
Women, N (%)	357 (65.5%)^b^	561 (46.0%)
Black race, N (%)	154 (28.3%)^b^	508 (41.7%)
Pittsburgh site, N (%)	243 (44.6%)^a^	464 (38.1%)
Body mass index, kg/m^2^	26.7 ± 4.8^b^	27.5 ± 4.7
Current smoker, N (%)	39 (7.2%)	116 (9.5%)
Alcohol > 1 drink/week, N (%)	257 (47.9%)	609 (50.7%)
≥ High school graduate, N (%)	440 (81.6%)^b^	872 (72.4%)
Fall history, N (%)	144 (26.6%)^a^	242 (19.9%)
Hypertension, N (%)	311 (57.0%)	695 (57.1%)
Cardiovascular disease, n (%)	91 (16.9%)	208 (17.3%)
Cerebrovascular disease, n (%)	45 (8.3%)	78 (6.5%)
Diabetes, N (%)	107 (19.7%)	264 (21.8%)
Cystatin C > 1.0, N (%)	236 (44.0%)	556 (46.0%)
Poor vision, N (%)	12 (2.2%)	25 (2.1%)
CES-D score	4.6 ± 5.2	4.5 ± 5.2
3MSE score	91.7 ± 6.9^b^	90.3 ± 8.1
Knee or leg pain, N (%)	181 (33.3%)	379 (31.2%)
1.4 g Monofilament insensitivity, N (%)	207 (38.8%)	421 (35.1%)
6-m Gait speed, m/second	1.2 ± 0.2	1.2 ± 0.2
Number of medications		
1998/99 clinic visit	3.5 ± 2.7^a^	3.0 ± 2.6
1999/00 clinic visit	3.7 ± 2.9^b^	3.3 ± 2.7
2001/02 clinic visit	4.3 ± 3.1^b^	3.7 ± 2.7
2002/03 clinic visit	4.5 ± 3.2^b^	4.0 ± 2.9
2004/05 clinic visit	5.1 ± 3.3^a^	4.6 ± 3.2
2006/07 clinic visit	5.7 ± 3.3^a^	5.0 ± 3.5
2007/08 clinic visit	5.9 ± 3.7^a^	5.3 ± 3.6
Use of ≥1 FRID, N (%)		
1998/99 clinic visit	126 (23.1%)^b^	201 (16.5%)
1999/00 clinic visit	142 (26.0%)^b^	220 (18.1%)
2001/02 clinic visit	157 (28.8%)^b^	252 (20.7%)
2002/03 clinic visit	158 (29.0%)^b^	270 (22.1%)
2004/05 clinic visit	122 (22.4%)^b^	232 (19.0%)
2006/07 clinic visit	109 (20.0%)^b^	211(17.3%)
2007/08 clinic visit	102 (18.7%)^b^	196 (16.1%)

*CES-D* Center for Epidemiologic Studies Depression Scale score, range is 0 to 60; a score of 16 points or more is considered depressed, *3MSE* Modified Mini-Mental State Examination, range is 0 to 100; lower scores represent lower levels of mental competency, *FRID* Fall risk increasing drugs

^a^*P* < 0.05, ^b^ < 0.01 for fall injury vs. no fall injury

In the univariate Cox model, participants with persistent polypharmacy had an increased fall injury risk than those with non-persistent polypharmacy (HR: 1.29 [1.05, 1.59]). After adjusting for age, sex, race, study site, alcohol use, BMI, 1.4-g monofilament insensitivity, fall history, the HR of persistent polypharmacy was 1.31 (1.06, 1.63) (Table 2). Compared to participants who had non-persistent polypharmacy without FRID use, those with persistent polypharmacy and FRID use had a significantly increased fall injury risk (HR: 1.48 [1.10, 2.00]). Compared to non-persistent polypharmacy without FRID use, HRs for persistent polypharmacy without FRID use (HR: 1.22 [0.93, 1.60]) and non-persistent polypharmacy with FRID use (HR: 1.08 [0.77, 1.51] did not show an association with fall injury risk. Results of sensitivity analyses were consistent with the primary analyses after including those with intermittent polypharmacy in the referent group (Supplementary Table S2). Most of the results were consistent with the primary analyses when cut-points of 7 to 10 medications were used to define persistent polypharmacy. In addition, there was also a noticeable jump in the HR for fall injury when going from a cut-point of 5 to a cut-point of 6. This finding supports the criteria of 6 prescription medications as an appropriate cut-point to distinguish the effect of persistent polypharmacy [6]. (Supplementary Table S3). The results without LOCF were consistent with primary analyses (Supplementary Table S2).

**Table 2:** Adjusted hazard ratios of persistent polypharmacy and FRID on fall injury in the primary analysis

	Model 1		Model 2	
	HR (95% CI)	*p*-value	HR (95% CI)	*p*-value
PP vs. non-PP	1.31(1.06,1.63)	0.014		
Joint-effect of PP and FRID use (reference: Non-PP without FRID use)				
PP with FRID use			1.48(1.10,2.00)	0.010
PP without FRID use			1.22(0.93,1.60)	0.155
Non-PP with FRID use			1.08(0.77,1.51)	0.659
Body mass index, per 1 kg/m^2^	0.98(0.96,1.00)	0.060	0.98(0.96,1.00)	0.061
Age, per 1 year	1.03(1.00,1.07)	0.046	1.03(1.00,1.07)	0.048
Women vs. men	2.09(1.71,2.56)	<.0001	2.08(1.70,2.54)	<.0001
Blacks vs. whites	0.64(0.51,0.79)	<.0001	0.64(0.51,0.80)	<.0001
Pittsburgh site vs. Memphis site	1.54(1.27,1.87)	<.0001	1.55(1.27,1.88)	<.0001
Fall history vs. no history	1.29(1.05,1.59)	0.015	1.29(1.05,1.59)	0.017
1.4 g Monofilament insensitivity	1.22(1.01,1.47)	0.040	1.22(1.01,1.47)	0.042

Education (<high school (HS), HS graduate, or postsecondary), self-reported physical activity (kcal/kg per week spent walking, climbing stairs and exercise), self-reported smoking status (current, past, never), alcohol > 1 drink/week, self-reported cardiovascular diseases, poor vision, knee pain for > 1 month, leg pain when walking, diagnosed and/or treated hypertension and diabetes, CES-D score, Modified Mini-Mental State Examination (3MSE) scores, cystatin C level > 1 mg/dL, gait speed over 6 m were added to the model in a stepwise manner and subsequently removed at a P > 0.10

*FRID* Fall risk increasing drugs, *PP* Persistent polypharmacy

## Discussion

In this prospective study of community-dwelling older adults with Medicare FFS, we found an increased risk of treated fall injury with persistent polypharmacy, particularly when combined with use of FRID. In our findings, older adults with persistent polypharmacy and FRID use had nearly 50% higher fall injury risk compared to those who had non-persistent polypharmacy without FRID use. To our knowledge, our study was the first to suggest a synergic effect of polypharmacy and FRID on the risk of fall injuries treated in both inpatient and outpatient settings.

The contribution of FRID use to the association of persistent polypharmacy and fall injury risk has not been well investigated. Previous studies in community-dwelling older adults showed that polypharmacy was associated with inpatient treated fall injuries after accounting for the confounding effect of FRID, but did not report the contribution of FRID use to the association [9, 32, 33]. However, our study observed a possible synergic effect from polypharmacy and FRID use on fall injury risk. The varying definitions for FRID use makes it difficult to compare across previous work and is a limitation in the field. Our FRID definition was modified based on the Beer’s Criteria commonly used in the US [17–19]; while previous studies used medication lists from their national fall prevention guidelines or STOPP/Fall criteria which is widely used in European countries [9, 32–34]. Our definition was tailored to the Health ABC population, excluding antihypertensives and some anticholinergics due to non-significant association with fall risk shown in previous published Health ABC findings [16, 22]. The other major difference in our study vs. previous studies is the inclusion of outpatient fall injuries (i.e. falls and fall injuries treated in outpatient clinics, emergency departments, or other outpatient settings) which are estimated to constitute around 57% of fall injuries requiring medical care [35]. Our results may suggest older adults with polypharmacy and FRID use were at higher risk of fall injuries leading to treatment in outpatient settings in addition to inpatient hospital settings [9, 32, 36]. Future studies need to carefully consider the appropriateness of certain medication classes in the FRID definition for their populations, as no standard definition exists.

Persistent polypharmacy is most likely the consequence of chronic multi-morbidities [36, 37]. Older adults with multi-morbidities may be more likely to receive any treatment, which could increase the likelihood of receiving treatment for fall injuries as well, especially for less severe injuries [38, 39]. In addition, older adults with multi-morbidities who also use FRID may experience increased fall risks related to chronic conditions such as osteoarthritis, vision loss, and peripheral nerve impairment [40–42], as well as from effects of FRID use such as sedation, dizziness, and postural sway [15, 43–45]. The adverse effects of FRID use may be more likely in older adults with multi-morbidities and/or polypharmacy possibly due to increasing pharmacodynamic sensitivity to FRID or high risk of drug-drug interactions [15, 43]. Further studies are needed to replicate our findings in other samples of older adults to assess the joint effect of polypharmacy and FRID use in fall injury.

A growing number of observational studies, including our study, showed polypharmacy appears to be a risk factor for fall injury in community-dwelling older adults [9, 32, 44]. Interventions are needed to reduce the risk for fall injuries in older adults with polypharmacy living in the community. However, several randomized trials demonstrated that single intervention targeting withdrawing medications, including FRID use, did not reduce fall injury risk significantly among older adults [46, 47]. Discrepant results between observational studies and randomized trials possibly suggest that single intervention for medications is insufficient for reducing fall risk as observational studies are not able to account for unmeasured factors, such as the severity of chronic conditions that is correlated with polypharmacy and increases fall injuries as well. In addition, previous randomized trials showed the difficulty of implementing medication withdrawal interventions among older adults using multiple medications [48, 49]. Besides medication withdrawal interventions, other evidence-based recommendations for fall prevention include exercise and multifaceted interventions which are customized based on initial assessments in various components, such as balance, gait, vision, postural blood pressure, medication, environment, cognition, and psychological health [50]. Studies have shown that community-dwelling older adults who present in ED with a fall often did not receive fall risk assessment and relevant interventions in accordance with guidelines, representing a missed opportunity to address their fall risk profile [51, 52]. Some randomized trials further supported that customized fall prevention interventions including medication management initiated in ED services reduced the risk of subsequent fall injuries by 43-65% [53, 54]. Therefore, clinicians in outpatient settings, especially in ED may need to consider tailoring fall prevention strategies in multiple aspects, in addition to medication management for community older adults with polypharmacy, such as promoting exercise, wearing safe shoes, improving safety in the home environment, and additional preventative efforts related to chronic multi-morbidities (e.g. diabetes, cardiovascular diseases, and dementia).

Tailoring fall interventions by demographic groups may need to be considered in clinical practice to improve the effectiveness of fall prevention. Our study, in accordance with previous studies, demonstrated that women and White individuals were more likely to have fall injuries compared to men and Black individuals, respectively [55–58]. Sex differences in fall injury risk, especially higher fracture risk in women, are well-established [55, 59]. Racial differences in fall injury risk may reflect differences in chronic diseases in late life, such as higher prevalence of osteoporosis in older White populations [60]. In our population, education level was not associated with risk of fall injury after adjusting for race in the final model, suggesting race group explained the association of education level and fall injury. Further studies are needed to delineate the underlying mechanisms for lower fall injury risk in non-White older adults and evaluate the effectiveness of fall interventions in racially diverse aging populations of men and women.

Our study has several strengths. A follow-up for nearly 8 years allowed examining of the temporal relationship between polypharmacy and incident fall injuries. The combined variable of persistent polypharmacy and FRID use over time may improve our understandings of the role of FRID in the relationship between polypharmacy and fall injury risk. Our focus on persistent polypharmacy may provide more comprehensive insights of real-life medication use compared to previous studies that focused on polypharmacy at a single time point [9, 32, 61]. Our medication inventory was based on participants’ use rather than prescription fill or pharmacy databases which may be less accurate for actual use. We used a validated aggregate diagnoses code algorithm to identify treated fall injuries in both inpatient and outpatient settings compared to previous studies including only inpatient fall injuries based on hospital admissions [8, 9, 32]. In addition, our participants had more chronic conditions and a higher prevalence and incidence of fall injury compared to older adults ≤70 years old [62], allowing us to study older adults at high risk for both polypharmacy and fall injuries. Moreover, the cohort study data combined with Medicare FFS claims enabled us to adjust for a rich set of health conditions and factors compared to other studies that only used claims-based covariates.

A limitation of our study is that medication inventories were done annually though not done at certain clinic visits and the LOCF method was used to estimate the missing data from annual visits. The prevalence of persistent polypharmacy may be overestimated since LOCF may misclassify those without persistent polypharmacy as with persistent polypharmacy. Imputation of medication use is not an ideal approach in older populations due to the relationship of missing data at clinic visits and poor health status [63]. However, our results showed no changes when analyzing the data without LOCF. Annual medication inventories did not allow us to address non-adherence issues which are prevalent among older adults with polypharmacy [64]. The fall injuries in our study were medically treated; therefore, less serious, non-treated fall injuries were not ascertained. We also could not observe complete claims for those with dual eligibility for Medicaid and/or veteran affairs health insurance. Thus, the rate of treated fall injury events may be underestimated. Our results should be reevaluated in non-PA or TN residents since reporting E-code may not be mandatory in some states [21]. Finally, our population is community-dwelling older adults with Medicare FFS eligibility who met the inclusion criteria of the Health ABC cohort and had at least two clinic visits to measure persistent polypharmacy. These inclusion criteria likely resulted in a healthier cohort compared to the general Medicare FFS population since those missing clinic visits generally had older age, more mobility limitation, and more cognitive impairment [63]. Also, our results may be not representative of older adults with other health care coverage or in non-community based settings such as skilled nursing facility (SNF) settings.

## Conclusions

Our study showed persistent polypharmacy was associated with increased fall injury risk treated in both inpatient and outpatient settings, particularly when combined with FRID use. These findings suggest that future studies should consider targeting medication use such as FRID use and other fall prevention efforts, such as altering behaviors or living environment, and tailoring interventions by sex and racial groups in older adults with persistent polypharmacy to decrease treated fall injuries not only in inpatient but also in outpatient settings.

## Supplementary Information

**Additional file 1: Supplementary Table S1.** Drug list of fall risk increasing drugs. **Supplementary Table S2.** Adjusted hazard ratios of persistent polypharmacy and FRID on fall injury in the sensitivity analyses. **Supplementary Table S3.** Adjusted hazard ratios for persistent polypharmacy with varied cut points for medication counts and fall injury risk.

## Data Availability

The data that supports the findings of this study are available from NIA but restrictions apply to the availability of these data, which were used under license for the current study, and so are not publicly available. Data are however available from the authors upon reasonable request and with permission of NIA.
