# Supplementary material for: Persistent polypharmacy and fall injury risk: the Health, Aging and Body Composition Study
Source: BMC Geriatr. 2021 Dec 15;21:710. doi: 10.1186/s12877-021-02695-9 (PMC8675466; doi:10.1186/s12877-021-02695-9)
Supplement: Supplementary file 1 — Additional file 1: Supplementary Table S1. Drug list of fall risk increasing drugs. Supplementary Table S2. Adjusted hazard ratios of persistent polypharmacy and FRID on fall injury in the sensitivity analyses. Supplementary Table S3. Adjusted hazard ratios for persistent polypharmacy with varied cut points for medication counts and fall injury risk. [file 12877_2021_2695_MOESM1_ESM.docx]

Supplementary Table S1

Drug list of fall risk increasing drugs (FRID)

| **Antidepressant ING CODES (28 ING)** | |
| --- | --- |
| **281604xx ANTIDEPRESSANTS-OTHER** | |
| 28160415 | Trazodone |
| 28160422 | Atomoxetine |
| 28160434 | Bupropion |
| 28160437 | Adenosyl-methionine |
| 28160458 | Venlafaxine |
| 28160461 | Sibutramine |
| 28160486 | Nefazodone |
| **281605xx ANTIDEPRESSANTS-MAO INHIB** | |
| 28160505 | Phenelzine |
| 28160520 | Selegiline |
| 28160521 | Rasagiline |
| 28160599 | Tranylcypromine |
| **281606xx ANTIDEPRESSANTS-TRI/TETRACYC** | |
| 28160601 | Amitriptyline |
| 28160602 | Imipramine |
| 28160617 | Mirtazapine |
| 28160650 | Trimipramine |
| 28160681 | Doxepin |
| 28160682 | Amoxapine |
| 28160688 | Clomipramine |
| 28160689 | Desipramine |
| 28160695 | Nortriptyline |
| 28160697 | Protriptyline |
| **281607xx ANTIDEPRESSANTS-SSRIS** | |
| 28160701 | Fluoxetine |
| 28160702 | Paroxetine |
| 28160703 | Sertraline |
| 28160704 | Fluvoxamine |
| 28160705 | Citalopram |
| 28160710 | Duloxetine |
| 28160711 | Escitalopram |
| **Antipsychotics ING CODES (16 ING)** | |
| **281608xx ANTIPSYCHOTICS-OTHER** | |
| 28160801 | Tybamate |
| 28160804 | Chlorprothixene |
| 28160807 | Hydroxyzine |
| 28160822 | Risperidone |
| 28160834 | Quetiapine |
| 28160836 | Olanzapine |
| 28160846 | Aripiprazole |
| 28160858 | Loxapine |
| **281609xx ANTIPSYCHOTICS-PHENOTHIAZINES** | |
| 28160906 | Fluphenazine |
| 28160909 | Perphenazine |
| 28160912 | Thioridazine |
| 28160913 | Trifluoperazine |
| 28160996 | Triflupromazine |
| **281610xx ANTIPSYCHOTICS-BUTYROPHENONES** | |
| 28161014 | Haloperidol |
| **562200xx ANTIEMETICS** | |
| 56220089 | CHLORPROMAZINE |
| 56220096 | PROCHLORPERAZINE |
| **ANXIOLYTICS-Benzodiazepine ING CODES (11 ING)** | |
| **282402xx ANXIOLYTICS-BENZODIAZEPINES** | |
| 28240202 | Chlordiazepoxide |
| 28240205 | Diazepam |
| 28240206 | Flurazepam |
| 28240212 | Clonazepam |
| 28240215 | Oxazepam |
| 28240216 | Estazolam |
| 28240222 | Triazolam |
| 28240228 | Clorazepate |
| 28240231 | Temazepam |
| 28240232 | Alprazolam |
| 28240276 | Lorazepam |
| **ANALGESICS-OPIOID ING CODES (10 ING)** | |
| **280808xx ANALGESICS-OPIOID** | |
| 28080810 | Fentanyl |
| 28080813 | Hydromorphone |
| 28080817 | Meperidine |
| 28080818 | Methadone |
| 28080819 | Morphine |
| 28080840 | Propoxyphene |
| 28080854 | Tramadol |
| 28080881 | Opium |
| 28080883 | Oxycodone |
| 28080892 | Pentazocine |
| **HYPNOTICS AND SEDATIVES ING CODES (11 ING)** | |
| **282404xx ANXIOLYTICS-BARBITURATES** | |
| 28240404 | Butabarbital |
| 28240405 | Hexobarbital |
| 28240413 | Butalbital |
| **282408xx ANXIOLYTICS-OTHER** | |
| 28240820 | Meprobamate |
| 28240828 | Dichloralphenazone |
| 28240829 | Eszopiclone |
| 28240830 | Ramelteon |
| 28240834 | Zolpidem |
| 28240837 | Buspirone |
| 28240856 | Zaleplon |
| **120800xx PARASYMPATHOLYTIC AGENTS** | |
| 12080009 | SCOPOLAMINE |
| **Antiepileptic drugs ING CODES (13 ING)** | |
| **2812xxxx ANTICONVULSANTS** | |
| 28120405 | Phenobarbital |
| 28120407 | Primidone |
| 28120805 | Phenytoin |
| 28122007 | Carbamazepine |
| 28122010 | Magnesium sulfate |
| 28122011 | Oxcarbazepine |
| 28122015 | Valproic acid |
| 28122016 | Pregabalin |
| 28122020 | Gabapentin |
| 28122024 | Lamotrigine |
| 28122034 | Tiagabine |
| 28122035 | Topiramate |
| 28122040 | Levetiracetam |

This list include six classes of FRID (i.e. antiepileptic drugs, hypnotics/sedatives drugs, antipsychotics, antidepressants, benzodiazepines, and opioids) collected at clinic visits in 1997/98, 1998/99, 1999/2000, 2001/02, 2002/03, 2004/05, 2006/07, and 2007/08 and coded according to the Iowa Drug Information System

Supplementary Table S2

Adjusted hazard ratios of persistent polypharmacy and FRID on fall injury in the sensitivity analyses

|  | Cohort without LOCF, N=1,590 | | Cohort including intermittent polypharmacy users, N= 1,764 | |
| --- | --- | --- | --- | --- |
|  | Model 1 | Model 2 | Model 1 | Model 2 |
| PP | 1.29(1.01,1.64)* |  | 1.24(1.00,1.53)* |  |
| PP and FRID (Non-PP without FRID use as ref) |  |  |  |  |
| PP with FRID use |  | 1.47(1.04,2.08)* |  | 1.43(1.06,1.92)* |
| PP without FRID use |  | 1.18(0.87,1.59) |  | 1.16(0.89,1.52) |
| Non-PP with FRID use |  | 1.00(0.68,1.46) |  | 1.17(0.87,1.58) |
| Body mass index, per 1 kg/m^2^ | 0.98(0.96,1.00) | 0.98(0.96,1.00) | 0.98(0.96,1.00)* | 0.98(0.96,1.00)* |
| Age, per 1 year | 1.04(1.00,1.08)* | 1.04(1.00,1.08)* | 1.04(1.01,1.07)* | 1.04(1.01,1.07)* |
| Female vs. male | 2.06(1.66,2.55)* | 2.04(1.64,2.53)* | 1.99(1.65,2.40)* | 1.97(1.63,2.38)* |
| Blacks vs. whites | 0.63(0.50,0.81)* | 0.64(0.50,0.81)* | 0.66(0.54,0.81)* | 0.67(0.54,0.82)* |
| Pittsburgh site vs. Memphis site | 1.59(1.29,1.96)* | 1.60(1.30,1.97)* | 1.52(1.27,1.82)* | 1.53(1.28,1.84)* |
| Fall history vs. no history | 1.38(1.10,1.71)* | 1.37(1.10,1.71)* | 1.26(1.03,1.54)* | 1.26(1.03,1.53)* |
| 1.4g Monofilament insensitivity | 1.22(1.00,1.49) | 1.22(1.00,1.49) | 1.18(0.99,1.40)* | 1.17(0.98,1.40) |

Model 1 includes persistent polypharmacy as the primary independent variable. Model 2 includes the combined variable of PP and FRID as the primary independent variable. In both models, education, self-reported physical activity (kcal/kg per week spent walking, climbing stairs and exercise), self-reported smoking status (current, past, never), alcohol > 1 drink/week, self-reported cardiovascular diseases, poor vision, knee pain for > one month, leg pain when walking, diagnosed and/or treated hypertension and diabetes, CES-D score, Modified Mini-Mental State Examination (3MSE) scores, cystatin C level > 1 mg/dL, gait speed over 6m and quadriceps strength were added to the model in a stepwise manner and subsequently removed at a P >0.10.

FRID: fall risk increasing drugs; PP: persistent polypharmacy

Supplementary Table S3

Adjusted hazard ratios for persistent polypharmacy with varied cut points for medication counts and fall injury risk

| Cut points for medication counts | Model 1 | Model 2  (Non-persistent polypharmacy without FRID use as ref) | | |
| --- | --- | --- | --- | --- |
|  | Persistent polypharmacy | Persistent polypharmacy with FRID use | Persistent polypharmacy without FRID use | Non-persistent polypharmacy without FRID use |
| ≥2 vs. < 2 | 1.11(0.89,1.38) | 1.28(0.96,1.71) | 1.09(0.87,1.38) | 2.27(0.92,5.58) |
| ≥3 vs. < 3 | 1.10(0.90,1.33) | 1.26(0.96,1.66) | 1.05(0.85,1.31) | 1.29(0.68,2.44) |
| ≥4 vs. < 4 | 1.15(0.94,1.39) | 1.30(0.99,1.71) | 1.09(0.88,1.36) | 1.17(0.72,1.89) |
| ≥5 vs. < 5 | 1.15(0.94,1.40) | 1.28(0.96,1.70) | 1.12(0.88,1.42) | 1.25(0.86,1.81) |
| ≥6 vs. < 6 | 1.31(1.06,1.63)* | 1.48(1.10,2.00)* | 1.22(0.93,1.60) | 1.08(0.77,1.51) |
| ≥7 vs. < 7 | 1.27(0.99,1.62) | 1.46(1.03,2.06)* | 1.17(0.84,1.63) | 1.14(0.85,1.53) |
| ≥8 vs. < 8 | 1.40(1.05,1.87)* | 1.61(1.08,2.41)* | 1.29(0.87,1.92) | 1.15(0.88,1.51) |
| ≥9 vs. < 9 | 1.51(1.06,2.14)* | 1.95(1.22,3.11)* | 1.23(0.74,2.05) | 1.13(0.88,1.47) |
| ≥10 vs. < 10 | 1.58(1.03,2.43)* | 1.86(1.04,3.33)* | 1.44(0.79,2.65) | 1.18(0.92,1.52) |

Model 1 includes persistent polypharmacy as the primary independent variable. Model 2 includes the combined variable of PP and FRID as the primary independent variable. Both models include age, sex, race, study site, BMI, history of falls, 1.4-g monofilament insensitivity as covariates. FRID: fall risk increasing drugs. *: P<0.05
